# Supplementary material for: Investigation of wear behaviour and surface analysis of a coated H13 material for friction drilling application
Source: PLoS One. 2025 Aug 22;20(8):e0328168. doi: 10.1371/journal.pone.0328168 (PMC12373206; doi:10.1371/journal.pone.0328168)
Supplement: Attachment — Submitted filename: Comments.docx [file pone.0328168.s003.docx]

**Title:** Investigation of Wear Behaviour and Surface Analysis of a Coated H13 Material for Friction Drilling Application

**Reviewer Comments & Authors Response**

**Reviewer 1**

| **Sl. No.** | **Reviewer Comments** | **Authors Response** |
| --- | --- | --- |
| 1 | The time considered for measurement of wear rate shown in figure 4 about 10 second is very short. Why author is selected such shorter period to evaluate the wear rate of the specimen? | Thanks for pointing out the mistake. The wear rate taken is for 10 Minutes only. It is already mentioned in chapter 2 experiment procedure. |
| 2 | Same for the coefficient of friction as shown in figure 5 about 10 second is very short. Why author is selected such shorter period? | Thanks for pointing out the mistake, the wear study has done for 10 minutes only.  Updated in manuscript |
| 3 | In Fig 5, The white line indicates the CoF and the red line indicates the displacement of the probe in a horizontal direction during testing. For untreated, heated and AlCrN samples both signals are exhibiting similar kind of trend. But for TiAlN why CoF is lowered drastically, can author explain physics behind this? | Figure updated, Thanks |
| 4 | Fig 11 b shows the formation of the bushing and the surface roughness, is there any technique which can be implemented to avoid or reduce the formation of the bushing? | In friction drilling, bushing formation can be reduced by optimizing speed, tool geometry, lubrication, and backing support—but bushing remains a key factor for thread engagement and joint strength. |
| 5 | While the references cited in the manuscript are relevant and reasonably up-to-date, it would strengthen the work to include a broader range of recent studies in the field to ensure comprehensive coverage of current advancements. <https://doi.org/10.3390/ma16041594> doi:10.1177/13506501231184304. <https://doi.org/10.1007/s42452-019-1152-6>. <https://doi.org/10.1007/s12046-020-1266-y>. | All the references cited in the manuscript, thanks for the support. |
